# Evaluation of the Arabin cervical pessary for prevention of preterm birth in women with a twin pregnancy and short cervix (STOPPIT-2): An open-label randomised trial and updated meta-analysis

**DOI:** 10.1371/journal.pmed.1003506

**Published:** 2021-03-29

**Authors:** Jane E. Norman, John Norrie, Graeme MacLennan, David Cooper, Sonia Whyte, Sue Chowdhry, Sarah Cunningham-Burley, Xue W. Mei, Joel B. E. Smith, Andrew Shennan, Stephen C. Robson, Steven Thornton, Mark D. Kilby, Neil Marlow, Sarah J. Stock, Phillip R. Bennett, Jane Denton, on behalf of the STOPPIT-2 collaborative group

**Affiliations:** 1 Faculty of Health Sciences, University of Bristol, Bristol, United Kingdom; 2 Edinburgh Clinical Trials Unit, University of Edinburgh, Edinburgh, United Kingdom; 3 Centre for Healthcare Randomised Trials, University of Aberdeen, Aberdeen, United Kingdom; 4 Tommy’s Centre for Maternal and Fetal Health, MRC Centre for Reproductive Health, University of Edinburgh, Edinburgh, United Kingdom; 5 Usher Institute, University of Edinburgh, Edinburgh, United Kingdom; 6 Nuffield Department of Population Health, University of Oxford, Oxford, United Kingdom; 7 NIHR Oxford Biomedical Research Centre, Oxford, United Kingdom; 8 Department of Women’s and Children’s Health, King’s College London, London, United Kingdom; 9 Institute of Cellular Medicine, University of Newcastle, Newcastle, United Kingdom; 10 Barts and The London School of Medicine and Dentistry, Queen Mary University of London, London, United Kingdom; 11 Fetal Medicine Centre, Birmingham Women’s and Children’s NHS Foundation Trust and College of Medical and Dental Sciences, University of Birmingham, Birmingham, United Kingdom; 12 Elizabeth Garrett Anderson Institute for Women’s Health, University College London, London, United Kingdom; 13 Institute for Reproductive and Developmental Biology, Imperial College London, London, United Kingdom; 14 Multiple Births Foundation, London, United Kingdom; Cambridge University, UNITED KINGDOM

## Abstract

**Background:**

Preterm-labour-associated preterm birth is a common cause of perinatal mortality and morbidity in twin pregnancy. We aimed to test the hypothesis that the Arabin pessary would reduce preterm-labour-associated preterm birth by 40% or greater in women with a twin pregnancy and a short cervix.

**Methods and findings:**

We conducted an open-label randomised controlled trial in 57 hospital antenatal clinics in the UK and Europe. From 1 April 2015 to 14 February 2019, 2,228 women with a twin pregnancy underwent cervical length screening between 18 weeks 0 days and 20 weeks 6 days of gestation. In total, 503 women with cervical length ≤ 35 mm were randomly assigned to pessary in addition to standard care (*n =* 250, mean age 32.4 years, mean cervical length 29 mm, with pessary inserted in 230 women [92.0%]) or standard care alone (*n =* 253, mean age 32.7 years, mean cervical length 30 mm). The pessary was inserted before 21 completed weeks of gestation and removed at between 35 and 36 weeks or before birth if earlier. The primary obstetric outcome, spontaneous onset of labour and birth before 34 weeks 0 days of gestation, was present in 46/250 (18.4%) in the pessary group compared to 52/253 (20.6%) following standard care alone (adjusted odds ratio [aOR] 0.87 [95% CI 0.55–1.38], *p =* 0.54). The primary neonatal outcome—a composite of any of stillbirth, neonatal death, periventricular leukomalacia, early respiratory morbidity, intraventricular haemorrhage, necrotising enterocolitis, or proven sepsis, from birth to 28 days after the expected date of delivery—was present in 67/500 infants (13.4%) in the pessary group compared to 76/506 (15.0%) following standard care alone (aOR 0.86 [95% CI 0.54–1.36], *p =* 0.50). The positive and negative likelihood ratios of a short cervix (≤35 mm) to predict preterm birth before 34 weeks were 2.14 and 0.83, respectively. A meta-analysis of data from existing publications (4 studies, 313 women) and from STOPPIT-2 indicated that a cervical pessary does not reduce preterm birth before 34 weeks in women with a short cervix (risk ratio 0.74 [95% CI 0.50–1.11], *p =* 0.15). No women died in either arm of the study; 4.4% of babies in the Arabin pessary group and 5.5% of babies in the standard treatment group died in utero or in the neonatal period (*p =* 0.53). Study limitations include lack of power to exclude a smaller than 40% reduction in preterm labour associated preterm birth, and to be conclusive about subgroup analyses.

**Conclusions:**

These results led us to reject our hypothesis that the Arabin pessary would reduce the risk of the primary outcome by 40%. Smaller treatment effects cannot be ruled out.

**Trial registration:**

ISRCTN Registry ISRCTN 02235181.

ClinicalTrials.gov NCT02235181.

## Abstract 2

Jane Norman and co-workers report an evaluation of a cervical pessary for prevention of preterm birth in women with a short cervix bearing twins.

## Author summary

**Why was this study done?:**

- Preterm-labour-associated preterm birth is common in twin pregnancy, and is an important cause of perinatal mortality and morbidity.
- There is controversy on the clinical effectiveness and cost-effectiveness of the Arabin pessary for the prevention of preterm birth in women with a twin pregnancy and a short cervix.

**What did the researchers do and find?:**

- We randomised 503 women with a cervical length of 35 mm or less to either standard care (according to National Institute for Health and Care Excellence [NICE] guidelines) or standard care and an Arabin pessary, inserted before 21 weeks gestation.
- The primary obstetric outcome was spontaneous onset of labour and birth before 34 weeks 0 days of gestation; the primary neonatal outcome was a composite adverse outcome.
- We found no differences in rates of the primary obstetric and neonatal outcomes between the 2 groups.

**What do these findings mean?:**

- Our findings indicate that any reduction in preterm-labour-associated preterm birth conferred by the Arabin pessary is less than 40%.
- Our findings indicate that a cervical length scan between 18 weeks 0 days and 20 weeks 6 days in women with a twin pregnancy and using a threshold length of 35 mm was ineffective at ruling in or ruling out preterm-labour-associated preterm birth.

## Introduction

Multiple pregnancy accounts for around 3% of births worldwide, the majority being twin pregnancies. Preterm birth is significantly more common in twins [1], and hence twin pregnancies are associated with higher rates of perinatal death and morbidity and higher healthcare costs compared to singleton pregnancies.

Three strategies have been trialled to determine if they prevent preterm birth of twins, but none have proven effective. Progesterone appears ineffective following the most recent systematic review (2017) [1], although there is controversy around this. Cervical cerclage may have a role in women with a very short cervix (cervical length of <15 mm), or in the presence of a dilated cervix, but is ineffective overall [2]. The placement of a silicone pessary around the cervix, the Arabin pessary, has also been advocated. Initial studies in women with a singleton pregnancy suggested a strong treatment effect in the prevention of preterm birth in women with singleton pregnancy and a short cervix (odds ratio [OR] 0.18 [95% CI 0.08–0.37], *p <* 0.001) [3]. However, a recent meta-analysis suggests that a cervical pessary has no impact on preterm birth prevention in these women (relative risk 0.80 [95% CI 0.43–1.49], *p =* 0.48) [4]. In women with twin pregnancy, there have been 3 studies. Results for 1 of 2 studies [5,6] including women with a short cervix, and a subgroup of women with a short cervix in another study [7], suggested that pessary placement reduces preterm birth. In contrast, in 2 studies of unselected women with twin pregnancy, there was no overall effect [7,8]. If effective, the Arabin pessary would potentially have wide applicability for the prevention of prematurity in twin pregnancies in women with a short cervix. The device itself is relatively inexpensive (current UK retail price £48) and can be inserted as an outpatient procedure, and side effects are reported to be ‘acceptable’ [7]. However, to date, trial results are conflicting, and sample sizes modest, and include results from a post hoc determination of cervical length threshold [7].

STOPPIT-2 is an open randomised controlled trial of the Arabin pessary to prevent preterm birth in twin pregnancy in woman with a short cervix (ISRCTN02235181, NCT02235181). The acronym is a reference to the STOPPIT study [9], which tested the effectiveness of progesterone for the prevention of preterm birth in twins. STOPPIT-2 was designed to test the hypothesis that, compared to standard treatment alone, the Arabin cervical pessary and standard care reduces the frequency of spontaneous labour associated with preterm birth in women with a twin pregnancy and cervical length ≤ 35 mm (‘short’ cervix), thus reducing adverse neonatal outcomes and healthcare costs. We also explored acceptability to pregnant women and a priori the effectiveness in 2 subgroups: women with a cervical length ≤ 25 mm, and women with a monochorionic twin pregnancy.

## Methods

### Study design and participants

STOPPIT-2 was an open-label randomised controlled superiority trial conducted in 57 antenatal clinics caring for women with multiple pregnancy at 56 UK NHS hospitals and 1 hospital in Belgium. The study protocol is published [10].

Participants were women with an uncomplicated twin pregnancy attending for antenatal care during the recruitment period of the study. The study was in 2 phases—(i) screening for eligibility by ultrasound and (ii) randomisation to treatment. All of the following inclusion criteria were required for eligibility for both the screening and treatment phases of the study: twin pregnancy (monochorionic or dichorionic), known chorionicity (as defined by first trimester ultrasound screening), current gestation ≤20 weeks + 6 days (as established by scan at ≤16 weeks according to NICE guidelines), age 16 years or older, and willingness to participate in both the screening and treatment phase of the study. Women with a short cervix (intended to be at or below the 30th centile) identified during the screening phase were eligible for inclusion in the treatment phase. For the first 6 months of the trial, the cervical length threshold for inclusion used was ≤30 mm, but this was changed to ≤35 mm after 6 months when it became clear that the 30th centile of our target population was 35 mm [10]. Women with bulging fetal membranes at the time of pessary insertion or with suspected or proven rupture of the fetal membranes at the time of pessary insertion were excluded. All cervical length measurements were performed using transvaginal ultrasound by a sonographer (radiographer, midwife, or obstetrician) who had undergone training through the CLEAR programme (https://clear.perinatalquality.org/) or the Fetal Medicine Foundation training programme (https://fetalmedicine.org/). All participants provided written informed consent for both phases of the study on initial recruitment. Women who were eligible to enter the treatment phase of the study were offered the opportunity to withdraw before randomisation.

### Randomisation and masking

Following written informed consent, participants who fulfilled the criteria for the treatment phase of the study were allocated to 1 of 2 groups in a 1:1 ratio: Arabin pessary plus standard care or standard care alone. Randomisation was carried out by entering patient details into a web portal at the Centre for Healthcare Randomised Trials (CHaRT) at University of Aberdeen; treatment allocation was then assigned by computer. The allocation sequence employed minimisation with a random element (20%) using the variables study centre and chorionicity (mono- or dichorionic).

Women were enrolled by a member of the investigator team responsible for recruitment, pessary insertion, and outcome data collection at each site. It was not considered possible to mask any of the participants, caregivers, or those collecting outcome data to treatment allocation.

### Procedures

#### Screening phase

Participating women had a transvaginal ultrasound measurement of cervical length performed between 18 weeks 0 days and 20 weeks 6 days of gestation by an accredited sonographer.

#### Treatment phase

Women who had a cervical length of ≤35 mm were consented and allocated to standard care with or without Arabin pessary. Pessaries were inserted by an obstetrician as an outpatient procedure after the cervical length scan and before 21 weeks of gestation. Inserting obstetricians watched a training video on pessary insertion, were provided with written guidance on pessary management, and (at their discretion) practised pessary insertion on a model prior to first insertion. The written guidance on pessary management included the manufacturer’s guidance on choice of size and referred to a publication on this issue [11]. The pessary was left in situ until 35–36 weeks 6 days of gestation unless labour started or membranes ruptured, the woman asked for the pessary to be removed, or the supervising clinician recommended removal.

Women in both groups received standard care based on NICE guidelines for management of women with multiple pregnancy dependent upon chorionicity [12]. Women were reviewed at 4-weekly intervals, and any adverse effects recorded. Key primary and secondary outcomes were collected at the birth of the baby and in the neonatal period. Outcomes were abstracted from hospital notes and entered into a web-based database by trained staff, usually a midwife. There was no central adjudication of outcomes.

### Outcomes

The primary obstetric outcome was defined as birth before 34 completed weeks following the spontaneous onset of labour. Preterm pre-labour rupture of membranes <34 weeks with or without contractions was included in this definition of spontaneous onset of labour assuming birth occurred before 34 weeks; women with induction of labour or cesarean section before 34 weeks due to maternal or fetal conditions were not included in this definition of the primary outcome.

The primary neonatal outcome was a composite of adverse outcomes, including stillbirth or neonatal death, periventricular leukomalacia, early respiratory morbidity (defined as any need for supplemental oxygen > 30%, continuous positive airway pressure [CPAP], intratracheal ventilation, or surfactant replacement therapy within the first week after birth), intraventricular haemorrhage, necrotising enterocolitis, and proven sepsis, all measured up to 28 days after the expected date of delivery [10]. Miscarriages occurring at any time from recruitment were counted as stillbirths.

Key obstetric secondary outcomes were mean gestational age at delivery, any birth before 37 weeks of gestation, adverse events including infection and cervical trauma, acceptability of the pessary (determined by participant questionnaire), experience of the device throughout the study, and time of pessary removal. The frequency of each component of the primary composite neonatal outcome, birthweight, any deaths of live-born babies within the first 28 days, and discrete episodes of bloodstream or central nervous system infection (positive blood or cerebrospinal fluid culture, categorised by timing either within the first 72 hours or between 72 hours and discharge) were recorded.

For women screened but not randomised, we collected the frequency of birth before 34 weeks.

There were no changes to trial outcomes after commencement of the trial. A completed CONSORT checklist is provided (see S1 CONSORT Checklist).

### Statistical analysis

In a prospective UK cohort study [13], 35% of spontaneous deliveries occurred at <34 weeks. In the ProTWIN study [7], estimated relative risk was 0.6 (B.W.Mol personal communication based on a subgroup of participants). We calculated that a sample size of 500 women would have 94% power to detect a relative risk of 0.6 for the primary obstetric outcome. Even with imperfect compliance and losses to follow-up in each group of up to 20%, power is preserved at 85%. Based on the ProTWIN study [7], in which the neonatal adverse outcome rate was 24% and relative risk 0.6, this sample size would provide 97% power to detect such a difference in the composite neonatal outcome, in the absence of any adjustment for clustering; allowing for 20% loss to follow-up (as per the obstetric primary outcome) and a between-twins intraclass correlation of 0.5, the study would still have over 80% power for this neonatal outcome. However, we anticipated a lower outcome frequency because we recruited women with longer cervices compared to the relevant subgroup in the ProTWIN study. If the prevalence of the composite neonatal outcome was 18%, we estimated study power to be 88% at 5% significance. Taking all the above into account, we chose a sample size to give a minimum of 0.8 power for the neonatal outcome, which then gives 0.85 power for the obstetric outcome, given the assumptions (clustering and dropout).

The frequency of the 2 primary outcomes in the study groups was compared in an intention to treat analysis, using logistic regression with a fixed effect for the minimisation covariate chorionicity, and a random effect for centre, to derive ORs and 95% confidence intervals of treatment effect. There are a small number of missing data points (4 and 8 mothers and therefore 8 and 16 babies, respectively), and we have assumed ‘no event’ where there are missing data. We intended to use multinomial logistic regression for secondary outcomes with more than 1 category, and linear regression for continuous secondary outcomes, adjusting for chorionicity and clustering within twins. However, our planned 3-level linear regression model for the primary neonatal outcome (babies nested within mother nested within centre, adjusting for chorionicity) failed to converge: We therefore used standard logistic regression for the neonatal primary outcome, adjusting for chorionicity and clustering at the mother level. For primary outcomes, predefined subgroup analyses were performed in women with monochorionic pregnancies, cervical length ≤ 25 mm and cervical length ≤ 28 mm; for these statistical analyses, significance was set at the 1% level, and data are presented as 95% CIs. For the secondary outcomes and the subgroup analyses, significance was set at the 1% level, and to account for multiple testing, 99% CIs were obtained, but data are presented as 95% CIs. All analyses were performed in Stata 15. We also calculated likelihood ratios for delivery before 34 weeks of gestation for women with cervical length ≤ 35 mm.

An independent data monitoring committee (IDMC) oversaw the analysis. A detailed statistical analysis was created and signed off by the IDMC prior to study completion. The trial was registered with the ISRCTN registry under the reference number ISRCTN98835694 and also with ClinicalTrials.gov with the reference number NCT02235181.

#### Meta-analysis

On 28 November 2020, an electronic search of the database PubMed was performed for clinical trials in twin or higher multiple pregnancy using the terms cervical pessary AND preterm birth AND multiple pregnancy to identify randomised trials comparing a cervical pessary and standard care with standard care alone for the prevention of preterm birth in women with twin or multiple pregnancy. Studies were restricted to those published in English. There was no attempt to contact authors of unpublished studies. We extracted data on women with a short cervix (using the definition of short cervix relevant for each individual study) and contacted authors for additional information where outcomes for the short cervix subgroup were not available. Meta-analysis of these studies, together with the data from STOPPIT-2, was performed in Stata15 using the DerSimonian and Laird random effects model with the heterogeneity estimate obtained from the Mantel–Haenszel model. A sensitivity analysis was performed restricting the analysis to studies that used the Arabin pessary for the prevention of preterm birth.

### Ethics statement

Ethics approval was given by South East Scotland Research Ethics Committee 02 on 29 August 2014, reference 14/SS/1031, and in Belgium on 21 September 2016, reference S58820.

## Results

**Fig 1:**
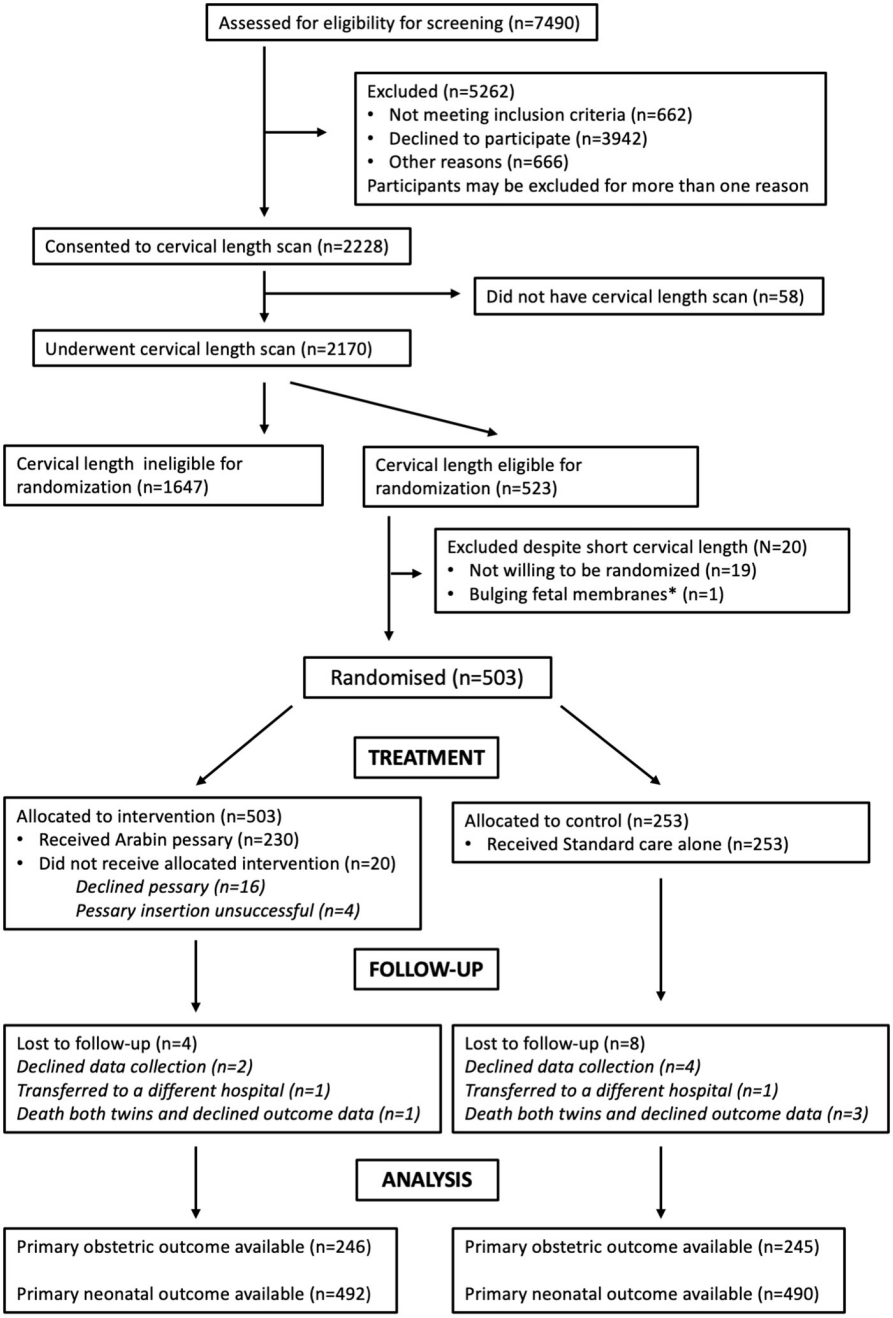
Participant flowchart. * Bulging fetal membranes noted de novo since qualifying cervical length scan.

In total, 2,228 women consented to cervical length screening between 1 April 2015 and 14 February 2019 (participants in the UK) or 13 December 2016 and 28 December 2018 (participants in Belgium), of whom 2,170 had a transvaginal scan. Of these, 523 were eligible for randomisation, and 503 agreed to be randomised into the treatment phase of the study, 250 to the intervention (Arabin pessary and standard care) and 253 to standard care alone (see participant flowchart Fig 1). The duration of pessary placement for individual women is shown in S1 Fig. The pessary size used for the majority of participants was 70 × 25 × 32 mm; a frequency table of pessary sizes is shown in S1 Table. The last participant visit was on 2 August 2019. Primary outcome data were available for 491/503 (97.6%) women; in the intervention and the control groups, respectively, 4 and 8 women were lost to follow-up or declined data collection but are included in the denominator for both the obstetric and neonatal outcomes. The 2 groups were well matched over a range of baseline measures (Table 1). The median (interquartile range) number of women randomised to the pessary group per centre was 3 (1–6).

**Table 1:** Baseline characteristics of randomised groups.

Characteristic	Arabin pessary, *N* = 250	Standard treatment, *N* = 253
Age (years)—mean (range)	32.4 (17, 51)	32.7 (17, 50)
Cervical length (mm)—mean (SD)	28.8 (5.8)	29.5 (5.1)
Minimum, maximum	3.0, 35.0	7.0, 35.0
Current smoker	21 (8.4%)	20 (7.9%)
Current alcohol	1 (0.4%)	3 (1.2%)
Obstetric history		
Previous livebirths		
0	150 (60.0%)	135 (53.4%)
1	60 (24.0%)	77 (30.4%)
2	17 (6.8%)	27 (10.7%)
3	12 (4.8%)	8 (3.2%)
4	7 (2.8%)	3 (1.2%)
5	3 (1.2%)	3 (1.2%)
6	1 (0.4%)	0 (0.0%)
Previous miscarriage		
No previous pregnancies	107 (42.8%)	99 (39.1%)
0	60 (24.0%)	65 (25.7%)
1	50 (20.0%)	49 (19.4%)
2	17 (6.8%)	29 (11.5%)
3	7 (2.8%)	6 (2.4%)
4	6 (2.4%)	1 (0.4%)
5	1 (0.4%)	3 (1.2%)
6	2 (0.8%)	1 (0.4%)
Medical conditions		
Hypertension	4 (1.6%)	8 (3.2%)
Insulin-dependent diabetes	2 (0.8%)	3 (1.2%)
Respiratory disease	11 (4.4%)	13 (5.1%)
Cardiac disease	5 (2.0%)	3 (1.2%)
Neurological disease	3 (1.2%)	3 (1.2%)
Skin condition	3 (1.2%)	3 (1.2%)
Thrombophilia	1 (0.4%)	3 (1.2%)
Current pregnancy		
Fetal anomaly scan—twin 1		
Normal	198 (79.2%)	209 (82.6%)
Defined abnormality	4 (1.6%)	2 (0.8%)
Uncertain abnormality	2 (0.8%)	1 (0.4%)
Not done	43 (17.2%)	40 (15.8%)
Fetal anomaly scan—twin 2		
Normal	199 (79.6)	211 (83.4)
Defined abnormality	0	0
Uncertain abnormality	3 (1.2%)	1 (0.4%)
Not done	43 (17.2%)	40 (15.8%)
Chorionicity		
Monochorionic diamniotic	50 (20.0%)	51 (20.2%)
Dichorionic diamniotic	200 (80.0%)	202 (79.8%)

Data are given as *n* (percent) unless otherwise indicated.

The primary obstetric outcome, the proportion of women with preterm delivery before 34 weeks following spontaneous onset of labour, was 46/250 (18.4%) in the Arabin pessary and standard care group and 52/253 (20.6%) in the standard care alone group (adjusted OR [aOR] 0.87 [95% CI 0.55–1.38], *p =* 0.54; Table 2). The proportion of babies with the primary composite neonatal outcome was 67 (13.4%) in the pessary and standard care group and 76 (15.0%) following standard care alone. The unadjusted OR for the primary neonatal outcome was 0.88 (95% CI 0.61–1.25; *p* = 0.46). Our planned 3-level linear regression model for the primary neonatal outcome (babies nested within mother nested within centre, adjusting for chorionicity) failed to converge: We therefore used standard logistic regression for the neonatal primary outcome, adjusting for chorionicity and clustering at the mother level, giving an aOR of 0.86 (95% CI 0.54–1.36; *p* = 0.52) (Table 3). The frequency of secondary obstetric outcomes (Table 4), secondary neonatal outcomes (Table 5), and safety outcomes (S2 Table) did not differ significantly between the pessary and standard care and the standard care alone groups. Post hoc tests of interaction did not identify any differential effect on obstetric or neonatal outcome by subgroup (S2 Fig). A time to event plot (a post hoc analysis) is shown in S3 Fig. Results of per protocol analyses of the primary obstetric outcome (Table 6) and the primary neonatal outcome (Table 7) were similar to those of the intention to treat analyses.

**Table 2:** Primary obstetric outcome and key subgroup analyses.

Outcome or subgroup	*n/N* (%) women with outcome		Odds ratio (95% CI)	Risk ratio (95% CI)	*p*-Value
	Arabin pessary, *N =* 250	Standard treatment, *N =* 253			
**Delivery before 34 weeks**	46 (18.4%)	52 (20.6%)	0.87 (0.55, 1.38)	0.88 (0.66, 1.16)	0.54
**Primary obstetric outcome by subgroup**					
Monochorionic pregnancy	10/50 (20.0%)	6/51 (11.8%)	1.57 (0.34, 7.18)	1.67 (0.46, 6.06)	0.44
Dichorionic pregnancy	36/200 (18.0%)	46/202 (22.8%)	0.77 (0.39, 1.50)	0.78 (0.52, 1.18)	0.31
Cervical length ≤ 28 mm	27/89 (30.3%)	23/71 (32.4%)	0.85 (0.33, 2.19)	0.94 (0.60, 1.48)	0.40
Cervical length > 28 mm	19/161 (11.8%)	29/182 (15.9%)	0.72 (0.31, 1.67)	0.71 (0.39, 1.30)	0.31
Cervical length ≤ 25 mm	17/58 (29.3%)	18/39 (46.2%)	0.50 (0.15, 1.63)	0.66 (0.39, 1.14)	0.13
Cervical length > 25 mm	29/192 (15.1%)	34/214 (15.9%)	0.93 (0.45, 1.94)	0.92 (0.58, 1.45)	0.80

For the obstetric outcome, the odds ratio shown is adjusted for chorionicity, with a random effect for centre, and uses a mixed effects model. The risk ratio is adjusted for chorionicity and uses a generalised linear model clustering on centre. The subgroup analyses also include a variable for the subgroup and the interaction between the pessary variable and the subgroup variable.

**Table 3:** Primary composite neonatal outcome, components, and key subgroup analyses.

Outcome or subgroup	*n/N* (%) babies with outcome		Odds ratio (95% CI)	Risk ratio (95% CI)	*p*-Value
	Arabin pessary, *N =* 500	Standard treatment, *N =* 506			
**Composite neonatal outcome**	67 (13.4%)	76 (15.0%)	0.86 (0.54,1.36)	0.88 (0.60, 1.31)	0.52
**Components of neonatal outcome**					
Stillbirth or neonatal death	22 (4.4%)	28 (5.5%)			
Periventricular leukomalacia	5 (1.0%)	1 (0.2%)			
Early respiratory morbidity	36 (7.2%)	46 (9.1%)			
Intraventricular haemorrhage	9 (1.8%)	6 (1.2%)			
Necrotising enterocolitis	2 (0.4%)	10 (2.0%)			
Proven sepsis	9 (1.8%)	4 (0.8%)			
**Primary neonatal outcome by subgroup**					
Monochorionic pregnancy	22/100 (22.0%)	13/102(12.7%)	1.89 (0.51, 7.00)	1.69 (0.50, 5.02)	0.21
Dichorionic pregnancy	45/400 (11.3%)	63/404 (15.6%)	0.67 (0.34, 1.34)	0.71 (0.39, 1.29)	0.14
Cervical length ≤ 28 mm	41/178 (23.0%)	28/142 (19.7%)	1.19 (0.47, 3.00)	1.15 (0.56, 2.38)	0.63
Cervical length > 28 mm	26/322 (8.1%)	48/364 (13.2%)	0.57 (0.24, 1.33)	0.61 (0.28, 1.31)	0.09
Cervical length ≤ 25 mm	29/116 (25.0%)	20/78 (25.6%)	1.04 (0.32, 3.33)	1.05 (0.44, 2.50)	0.93
Cervical length > 25 mm	38/384 (9.9%)	56/428 (13.1%)	0.70 (0.34, 1.46)	0.74 (0.38, 1.41)	0.21

For the neonatal outcome, the odds ratio is adjusted for chorionicity and clustering at the mother level using standard logistic regression. The risk ratio is adjusted for chorionicity and clustering on centre using a generalised linear model. Out of 491 mothers, 399 had no primary neonatal outcomes for either twin, 41 had a primary neonatal outcome for 1 twin, and 51 had at least 1 primary neonatal outcome for both twins. For 3 centres, the minimum number of neonatal events was 2 (2 centres) and the maximum was 18 (1 centre).

**Table 4:** Secondary obstetric outcomes.

**Outcome—mean (SD)**	**Arabin pessary,** ***N* = 250**	**Standard treatment,** ***N* = 253**	**Mean difference (95% CI)**	***p*-Value**
Gestational age at delivery (weeks)	34.8 (3.7) [*N =* 246]	34.5 (4.0) [*N =* 245]	0.2 (−0.6, 1.1)	0.50
Duration of labour stage 1 (minutes)	403.9 (510.8) [*N =* 81]	326.0 (255.5) [*N =* 81]	77.1 (−85.2, 239.4)	0.22
Duration of labour stage 2 (minutes)	80.0 (90.7) [*N =* 77]	101.1 (202.3) [*N =* 80]	−21.3 (−85.7, 43.1)	0.39
Duration of labour overall (minutes)	333.4 (485.1) [*N =* 123]	325.7 (439.9) [*N =* 117]	5.4 (−147.5, 158.3)	0.93
Duration of hospital stay (days)	5.5 (7.2) [*N =* 243]	5.6 (5.4) [*N =* 242]	−0.1 (−1.6, 1.4)	0.87
**Outcome—*n* (%)**	**Arabin pessary**	**Standard treatment**	**Chi**^**2**^	***p*-Value**
Method of delivery—twin 1			chi^2^(3) = 0.835	0.84
Spontaneous vaginal delivery	62 (24.8)	63 (24.9)		
Vaginal breech	3 (1.2)	4 (1.6)		
Forceps or ventouse	20 (8.0)	15 (5.9)		
Cesarean section	160 (64.0)	159 (62.8)		
Method of delivery—twin 2			chi^2^(3) = 3.338	0.34
Spontaneous vaginal delivery	48 (19.2)	45 (17.8)		
Vaginal breech	13 (5.2)	23 (9.1)		
Forceps or ventouse	15 (6.0)	12 (4.7)		
Cesarean section	169 (67.6)	162 (64.0)		
**Outcome—*n* (%)**	**Arabin pessary**	**Standard treatment**	**Odds ratio (95% CI)**	***p*-Value**
Births				
Before 28 + 0 weeks	17 (6.8)	24 (9.5)	0.67 (0.27, 1.64)	0.25
Before 32 + 0 weeks	35 (14.0)	41 (16.2)	0.83 (0.42, 1.63)	0.47
Before 34 + 0 weeks	62 (24.8)	66 (26.1)	0.90 (0.52, 1.57)	0.64
Before 37 + 0 weeks	158 (63.2)	161 (63.6)	0.95 (0.57, 1.58)	0.79
Births preceded by spontaneous onset of labour				
All births	61 (24.4)	71 (28.1)	0.82 (0.48, 1.41)	0.34
Before 28 + 0 weeks	13 (5.2)	19 (7.5)	0.64 (0.23, 1.77)	0.26
Before 32 + 0 weeks	26 (10.4)	32 (12.6)	0.79 (0.37, 1.68)	0.43
Before 34 + 0 weeks	37 (14.8)	46 (18.2)	0.77 (0.40, 1.47)	0.30
Before 37 + 0 weeks	56 (22.4)	66 (26.1)	0.81 (0.47, 1.41)	0.32
pPROM	12 (4.8)	4 (1.6)	1.95 (0.52, 7.34)	0.20
Incidence of birth before 34 + 0 weeks preceded by pPROM	8 (3.2)	3 (1.2)	1.61 (0.36, 7.14)	0.41
Adverse events				
Infection	12 (4.8)	10 (4.0)	1.25 (0.39, 3.95)	0.62
Haemorrhage	115 (46.0)	105 (41.5)	1.19 (0.73, 1.94)	0.35
Tachycardia	6 (2.4)	7 (2.8)	0.70 (0.12, 4.17)	0.61

*p-*Values are for proportion in Arabin pessary versus standard treatment group from linear regression analysis or using proportional odds analysis, both adjusting for chorionicity and centre. pPROM, preterm premature rupture of membranes.

**Table 5:** Secondary neonatal outcomes.

**Outcome—mean (SD) or median (minimum, maximum)**	**Arabin pessary**	**Standard treatment**	**Difference in means (95% CI)**	***p-*Value**
Birthweight (g)	2,170 (659) [*N =* 488]	2,142 (686) [*N =* 485]	27 (−120, 174)	0.64
Cord pH (venous)	7.3 (3.4, 7.8) [*N =* 212]	7.3 (3.3, 7.4) [*N =* 192]	0.0 (−0.1, 0.0)	0.52
Cord pH (arterial)	7.3 (7.0, 7.4) [*N =* 199]	7.3 (3.4, 8.3) [*N =* 177]	0.0 (−0.0, 0.1)	0.09
Apgar score at 1 minute	9.0 (0, 10) [*N =* 472]	9.0 (0, 10) [*N =* 470]	0.1 (−0.3, 0.6)	0.46
Apgar score at 5 minutes	9.0 (0, 10) [*N =* 468]	9.0 (0, 10) [*N =* 467]	0.1 (−0.3, 0.5)	0.54
Days of oxygen therapy	21.5 (32.9) [*N =* 36]	9.3 (15.0) [*N =* 45]	12.9 (−4.0, 29.8)	0.05
Level of care days	22.0 (27.5) [*N =* 245]	25.0 (31.8) [*N =* 225]	−4.3 (−13.0, 4.5)	0.21
Cumulative inpatient days	19.6 (41.3) [*N =* 244]	21.8 (44.9) [*N =* 244]	−2.2 (−12.3, 7.9)	0.29
**Outcome—*n* (%)**	**Arabin pessary,** ***N* = 500**	**Standard treatment,** ***N* = 506**	**Odds ratio (95% CI)**	***p-*Value**
Birthweight < 10th centile	104 (20.8)	97 (19.2)	1.09 (0.69, 1.72)	0.64
Received resuscitation	119 (23.8)	125 (24.7)	0.93 (0.57, 1.52)	0.71
Fetal or neonatal death within the first 28 days after birth	4 (0.8)	8 (1.6)	0.49 (0.07, 3.25)	0.33
Received surfactant	39 (7.8)	40 (7.9)	0.97 (0.45, 2.08)	0.92
Bronchopulmonary dysplasia	6 (1.2)	3 (0.6)	2.00 (0.24, 16.58)	0.40
Necrotising enterocolitis	2 (0.4)	10 (2.0)	0.20 (0.03, 1.50)	0.04
Discrete episodes of bloodstream or CNS infection	3 (0.6)	2 (0.4)	1.50 (0.14, 15.76)	0.66
Daily level of care				
Normal care	67 (13.4)	59 (11.7)	1.15 (0.61, 2.16)	0.56
Special care	208 (41.6)	197 (38.9)	1.09 (0.70, 1.69)	0.61
High dependency	87 (17.4)	108 (21.3)	0.76 (0.45, 1.28)	0.18
Intensive	72 (14.4)	72 (14.2)	1.00 (0.54, 1.82)	0.98
Rate of major adverse neonatal outcomes before discharge from hospital	121 (24.2)	128 (25.3)	0.92 (0.57, 1.50)	0.67

Data refer to all twins, with the 95% CIs and *p*-values adjusted for clustering within twins. CNS, central nervous system.

**Table 6:** Primary obstetric outcome and key subgroup analyses per protocol analysis.

Outcome or subgroup	*n/N* (%) women with outcome		Odds ratio (95% CI)	Risk ratio (95% CI)	*p-*Value
	Arabin pessary, *N =* 230	Standard treatment, *N =* 253			
**Delivery before 34 weeks**	44 (19.1%)	52 (20.6%)	0.87 (0.55, 1.38)	0.91 (0.69, 1.20)	0.50
**Primary obstetric outcome by subgroup**					
Monochorionic pregnancy	10/47 (21.3%)	6/51 (11.8%)	1.57 (0.34, 7.18)	1.78 (0.49, 6.47)	0.44
Dichorionic pregnancy	34/183 (18.6%)	46/202 (22.8%)	0.77 (0.39, 1.50)	0.80 (0.52, 1.22)	0.31
Cervical length ≤ 28 mm	26/85 (30.6%)	23/71 (32.4%)	0.85 (0.33, 2.19)	0.94 (0.59, 1.49)	0.40
Cervical length > 28 mm	18/145 (12.4%)	29/182 (15.9%)	0.72 (0.31, 1.67)	0.75 (0.41, 1.38)	0.31
Cervical length ≤ 25 mm	16/55 (29.1%)	18/39 (46.2%)	0.50 (0.15, 1.63)	0.65 (0.37, 1.15)	0.13
Cervical length > 25 mm	28/175 (16.0%)	34/214 (15.9%)	0.93 (0.45, 1.94)	0.97 (0.61, 1.54)	0.80

For the obstetric outcome, the odds ratio shown is adjusted for chorionicity, with a random effect for centre, and uses a mixed effects model. The risk ratio is adjusted for chorionicity and uses a generalised linear model clustering on centre. The subgroup analyses also include a variable for the subgroup and the interaction between the pessary variable and the subgroup variable.

**Table 7:** Primary composite neonatal outcome, components and key subgroup analyses–per protocol.

Outcome or subgroup	*n/N* (%) babies with outcome		Odds ratio (95% CI)	Risk ratio (95% CI)	*p-*Value
	Arabin pessary, *N =* 460	Standard treatment, *N =* 506			
**Composite neonatal outcome**	66 (14.3%)	76 (15.0%)	0.93 (0.58, 1.47)	0.94 (0.64, 1.40)	0.74
**Components of neonatal outcome**					
Stillbirth or neonatal death	22 (4.8%)	28 (5.5%)			
Periventricular leukomalacia	5 (1.1%)	1 (0.2%)			
Early respiratory morbidity	35 (7.6%)	46 (9.1%)			
Intraventricular haemorrhage	9 (2.0%)	6 (1.2%)			
Necrotising enterocolitis	2 (0.4%)	10 (2.0%)			
Proven sepsis	9 (2.0%)	4 (0.8%)			
**Primary neonatal outcome by subgroup**					
Monochorionic pregnancy	22/94 (23.4%)	13/102 (12.7%)	2.05 (0.55, 7.63)	1.80 (0.61, 5.33)	0.16
Dichorionic pregnancy	44/366 (12.0%)	63/404 (15.6%)	0.72 (0.36, 1.45)	0.76 (0.41, 1.38)	0.23
Cervical length ≤ 28 mm	41/170 (24.1%)	28/142 (19.7%)	1.25 (0.49, 3.16)	1.19 (0.58, 2.46)	0.54
Cervical length > 28 mm	25/290 (8.6%)	48/364 (13.2%)	0.61 (0.26, 1.45)	0.65 (0.30, 1.41)	0.14
Cervical length ≤ 25 mm	29/110 (26.4%)	20/78 (25.6%)	1.09 (0.34, 3.50)	1.08 (0.46, 2.57)	0.85
Cervical length > 25 mm	37/350 (10.6%)	56/428 (13.1%)	0.76 (0.36, 1.59)	0.79 (0.41, 1.52)	0.33

For the neonatal outcome, the odds ratio is adjusted for chorionicity and clustering at the mother level using standard logistic regression. The risk ratio is adjusted for chorionicity and clustering on centre using a generalised linear model.

### Acceptability

All women in the standard care alone arm adhered to the intervention, with no out-of-trial pessary insertions. The maximum potential duration of pessary placement for women who did not deliver preterm prior to pessary removal was between 91 and 133 days (dependent on gestational age at insertion). Of the 250 women allocated to Arabin pessary, 16 women declined pessary insertion post-randomisation, and in 4 women, insertion was attempted but unsuccessful. The duration of pessary placement was recorded for 217 women, with a median period of 105 days (interquartile range 81–113) (S1 Fig). Twenty-six of 230 (11.3%) women who had the pessary inserted asked to have it removed before the scheduled date of removal, largely due to discomfort from the pessary (median placement of 14 days [interquartile range 6–98]), and in a further 13 women (5.7%) the pessary fell out after a median 69 days (interquartile range 27–100).

Of women in whom a pessary was inserted, 158/234 (67.5%) found insertion painless or slightly uncomfortable, whereas the remainder found it uncomfortable, very uncomfortable, or the worst pain imaginable. Clinicians described the procedure as easy or moderately easy in 202/234 (86.3%) cases, and difficult, very difficult, or impossible in 21/234 (9.0%) cases (S3 Table). Once the pessary had been inserted, the majority of women reported feeling the pessary either never or less than once a week, and rarely found it uncomfortable or painful (S3 Table). Removal was considered painless or uncomfortable in 95/230 (41.3%) women and was described as easy or moderately easy in 134/230 cases (58.3%) by clinicians (S3 Table).

Cervical length profiles of women in the screening and randomisation phases of the study are shown in S4 Fig (note that the data in this figure are from the screened population and not the trial population). The positive and negative likelihood ratios of a short cervix (≤35 mm) to predict preterm birth before 34 weeks were 2.14 (95% CI 1.67–2.74) and 0.83 (95% CI 0.76–0.90), respectively (S4 Table). For the other cervical lengths (≤30 mmm, ≤28 mm, ≤25 mm, and ≤20 mm), negative likelihood ratios were all more than 0.8, and positive likelihood ratios ranged from 3.27 to 9.13.

### Meta-analysis

**Fig 2:**
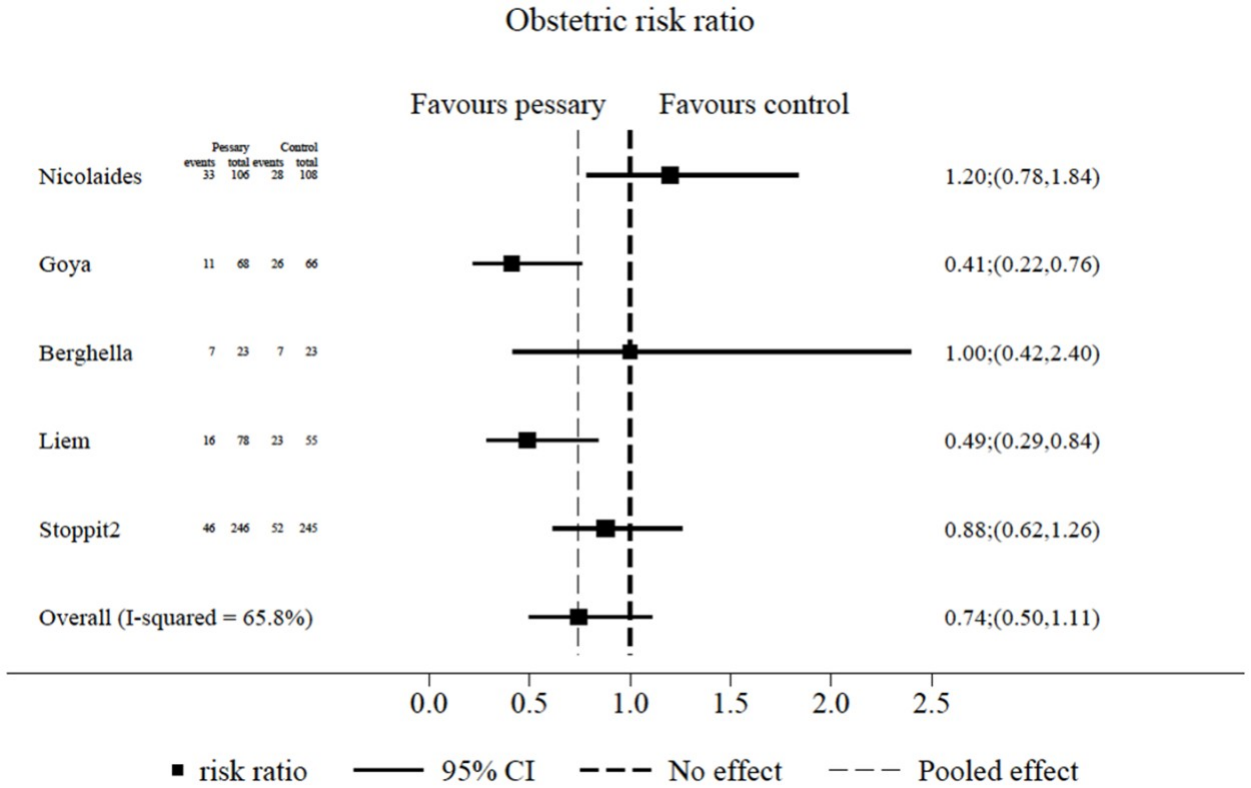
Meta-analysis of STOPPIT-2 and published data on the effectiveness of a cervical pessary in twin pregnancies in women with a short cervix in the prevention of preterm birth before 34 weeks gestation.

Electronic searches for clinical trials of the cervical pessary in twin or higher multiple pregnancy compared to placebo or standard care, and using the terms cervical pessary AND preterm birth AND multiple pregnancy, revealed 10 relevant publications. Of these, 3 publications were protocols and 3 were secondary analyses, leaving 4 published original studies [3,6–8]. Three studies used the Arabin cervical pessary [3,7,8]. One [6] used the Bioteque cup pessary (Bioteque, Fremont, CA, US), which is similar but not identical to the Arabin pessary. Alternative searches using the terms ‘twin pregnancy’ instead of ‘multiple pregnancy’ and ‘Arabin pessary’ instead of ‘cervical pessary’ did not identify any additional published trials. Meta-analysis of the data from STOPPIT-2 and from the 4 trials described above on women with a multiple/twin pregnancy and a short cervix (as defined by the paper authors) showed considerable heterogeneity amongst the studies (*I*^2^ 65.8%), with a risk ratio of birth before 34 weeks of gestation following Arabin pessary placement of 0.74 (95% CI 0.50–1.11; *p =* 0.15) (Fig 2). A sensitivity analysis restricting analysis to studies with the Arabin pessary (and therefore excluding the study with the Bioteque pessary) gave a risk ratio of 0.71 (95% CI 0.45–1.12; *p =* 0.14).

## Discussion

In this trial, insertion of an Arabin pessary did not reduce the incidence of either the primary obstetric outcome of preterm birth before 34 weeks of gestation following spontaneous onset of labour or the composite (or individual) adverse neonatal outcomes. Although the point estimate of the obstetric outcome might indicate benefit for those in the shortest cervix groups (≤25 mm or ≤28 mm), the point estimate of the neonatal outcome suggests the pessary could cause harm in these shorter cervix subgroups. None of these results reach statistical significance.

Our results accord with some [6,8] but not all [3,7] other efficacy or effectiveness studies in twin or higher multiple pregnancies. Our meta-analysis demonstrates considerable heterogeneity amongst existing published studies but shows an risk ratio of the effect of the pessary in preventing birth before 34 weeks of gestation is 0.74 (95% CI 0.50–1.11). In view of the heterogeneity of results of existing studies, the size of STOPPIT-2 (with the short cervix group being twice as big as the largest previously published study, and with a larger number of events than any previous study, to our knowledge), the use of a population threshold for cervical length in STOPPIT-2, and the ‘real world’ setting of STOPPIT-2, we believe our results (OR 0.87 [95% CI 0.55 to 1.38]) should prompt a change in practice for those clinicians currently using the pessary. A caveat is that we cannot exclude a benefit in a subgroup that is yet to be identified, particularly given many potential causes of short cervical length. Additionally, although the point estimate of the neonatal outcome shows harm in all subgroups of concern—monochorionic pregnancy, cervical length ≤ 28 mm, and cervical length ≤ 25 mm—the study is underpowered to be conclusive about subgroup analyses.

The strengths of our study are that treatment was allocated by central randomisation, and that the study used prespecified primary endpoints and followed a prespecified analysis plan. There was a low rate of loss to follow-up. Adherence was good; the vast majority of women in the pessary group had the pessary inserted (92%), and all pessaries were inserted by a clinician who had had specific training in this procedure. Only 26/230 (11.3%) women asked for the pessary to be removed prematurely: In these women, the median (SD) duration of adherence was 14 days. We achieved our prespecified sample size of 500 women randomised. A caveat is that we cannot exclude a small benefit (or harm): Although we achieved our prespecified sample size, there were fewer events than expected in the standard care alone group (52 and not 88). Hence the confidence intervals for our primary outcomes are larger than anticipated.

Insertion of the pessary had no effect on any secondary or safety outcome, and the majority of women found pessary insertion, their experience of the pessary during pregnancy, and pessary removal to be associated only with slight discomfort. Clinicians largely found pessary insertion easy, and placement was not possible in only 1.7% of women. We had intended to recruit women with cervical lengths at or below the 30th centile. We estimated this to a cervical length of 35 mm or below. Retrospective analysis showed that the 30th centile for the entire screening population was a cervical length of 36 mm.

The positive and negative likelihood ratios for a short cervix of ≤35 mm to predict preterm-labour-induced birth before 34 weeks were 2.14 and 0.83, respectively. Whilst these data suggest some association between short cervix and spontaneous preterm birth, the negative likelihood ratios for none of the 5 chosen cervical lengths achieved the threshold suggested for a moderately effective ‘rule out’ test [14]. In contrast, the positive likelihood ratios for cervical lengths of ≤20 mm and ≤25 mm for spontaneous preterm birth before 34 weeks were 9.13 and 7.82, respectively, values which confer moderate utility for a ‘rule in’ test [14]. We are confident that these likelihood ratios are close to the likelihood ratios in the population, given the size of our prospective cohort study [13,15]. Our data suggest that, as in singleton pregnancy, spontaneous preterm labour in twin pregnancy has multiple aetiologies, some but not all of which lead to cervical shortening in the second trimester of pregnancy.

Meta-analysis of our own and existing published studies confirms that the cervical pessary is not associated with a significant reduction in birth before 34 weeks of gestation in women with twin pregnancy.

### Study limitations

The main study limitations are the lack of power to show a smaller than 40% reduction in the primary obstetric outcome or to identify an effect in any of the cervical length subgroups, and the fewer than expected events in the standard care alone group, leading to wider confidence intervals than anticipated for the primary outcome.

Our findings suggest that the pessary should not be offered to women with twin pregnancy and a short cervix for the purpose of preventing preterm labour leading to preterm birth, and that routine cervical length scanning in otherwise uncomplicated twin pregnancies should not be introduced into routine clinical practice.

### Contributors

JEN conceived the study and wrote the first draft of the manuscript. JEN, JN, GM, DC, SW, SCB, JBES, AS, SCR, ST, MDK, NM, SJS, PRB, and JD wrote the study protocol, and contributed to study conduct and interpretation. DC, GM, JN, JBES, and XWM performed the primary analyses of the data All authors contributed to revisions of the manuscript and approved the final version prior to submission.

## Supporting information

S1 CONSORT Checklist (DOCX)

S1 Fig Duration of pessary placement for all trial participants. (TIF)

S2 Fig Post hoc tests of interaction by subgroup on obstetric or neonatal outcome. (TIF)

S3 Fig Time to event plot for the primary obstetric outcome. (TIF)

S4 Fig Cervical length profiles of women in the screening and randomisation phases of the study. (TIF)

S1 Table Frequency of use of pessary sizes for first pessary placement in the STOPPIT-2 study. (DOCX)

S2 Table Safety issues. (DOCX)

S3 Table Experiences of the pessary. (DOCX)

S4 Table Frequency of spontaneous birth before 34 weeks gestation for a variety of specified cervical lengths. (DOCX)

## Abbreviations

aOR: adjusted odds ratio
OR: odds ratio
