# Supplementary figures and images for: Evaluation of the Arabin cervical pessary for prevention of preterm birth in women with a twin pregnancy and short cervix (STOPPIT-2): An open-label randomised trial and updated meta-analysis
Source: PLoS Med. 2021 Mar 29;18(3):e1003506. doi: 10.1371/journal.pmed.1003506 (PMC8041194; doi:10.1371/journal.pmed.1003506)

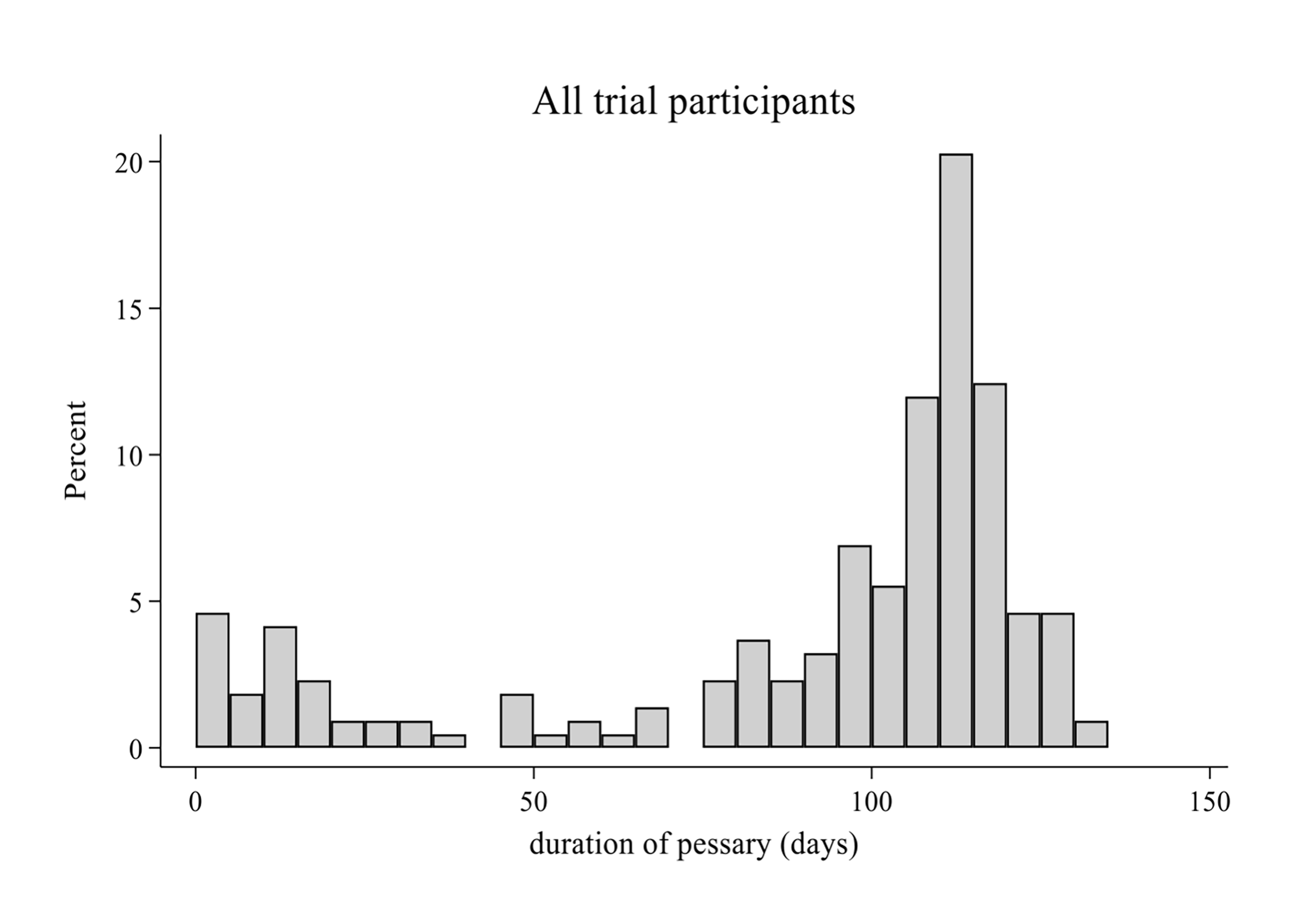

Supplement: S1 Fig — (TIF) [file pmed.1003506.s002.tif]

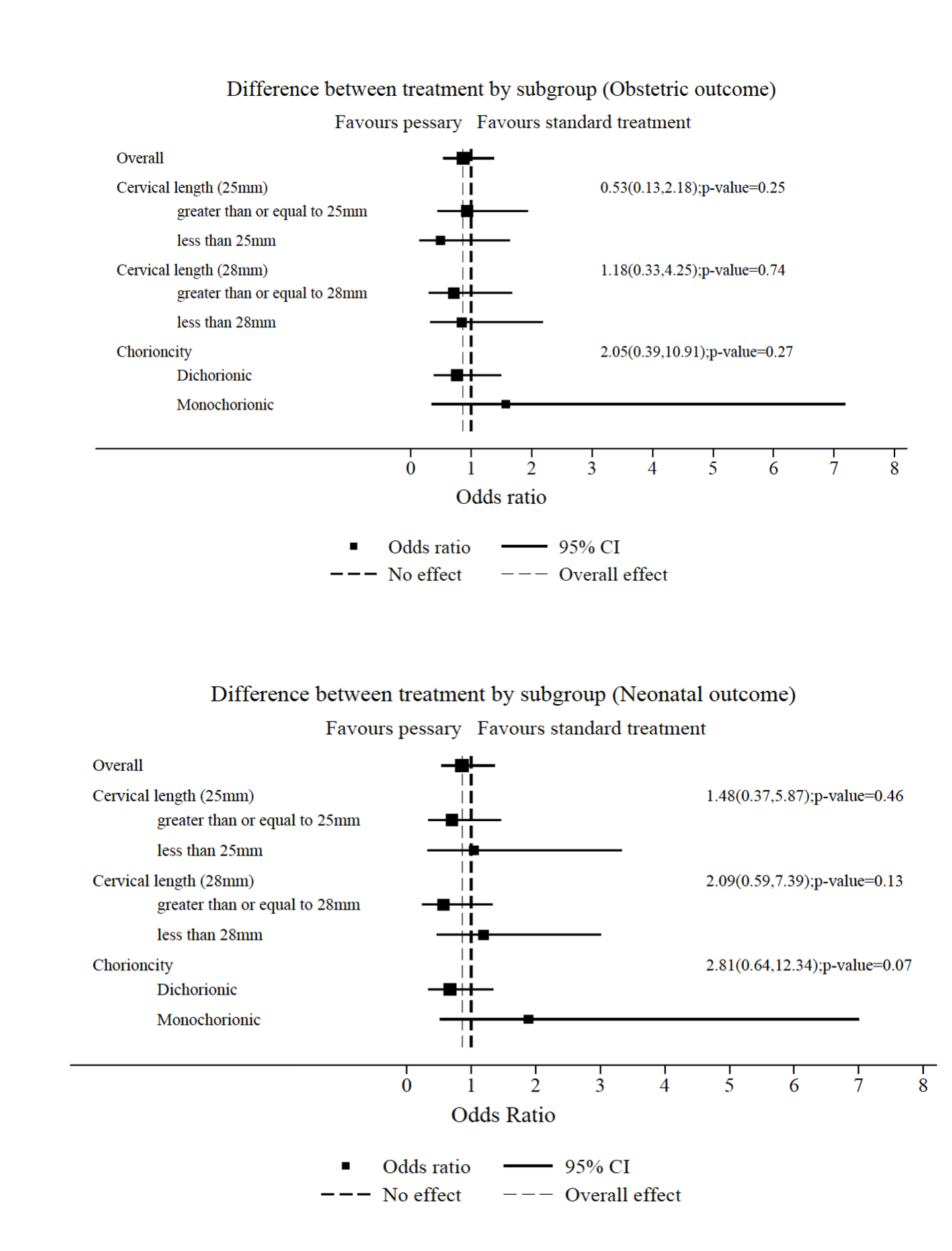

Supplement: S2 Fig — (TIF) [file pmed.1003506.s003.tif]

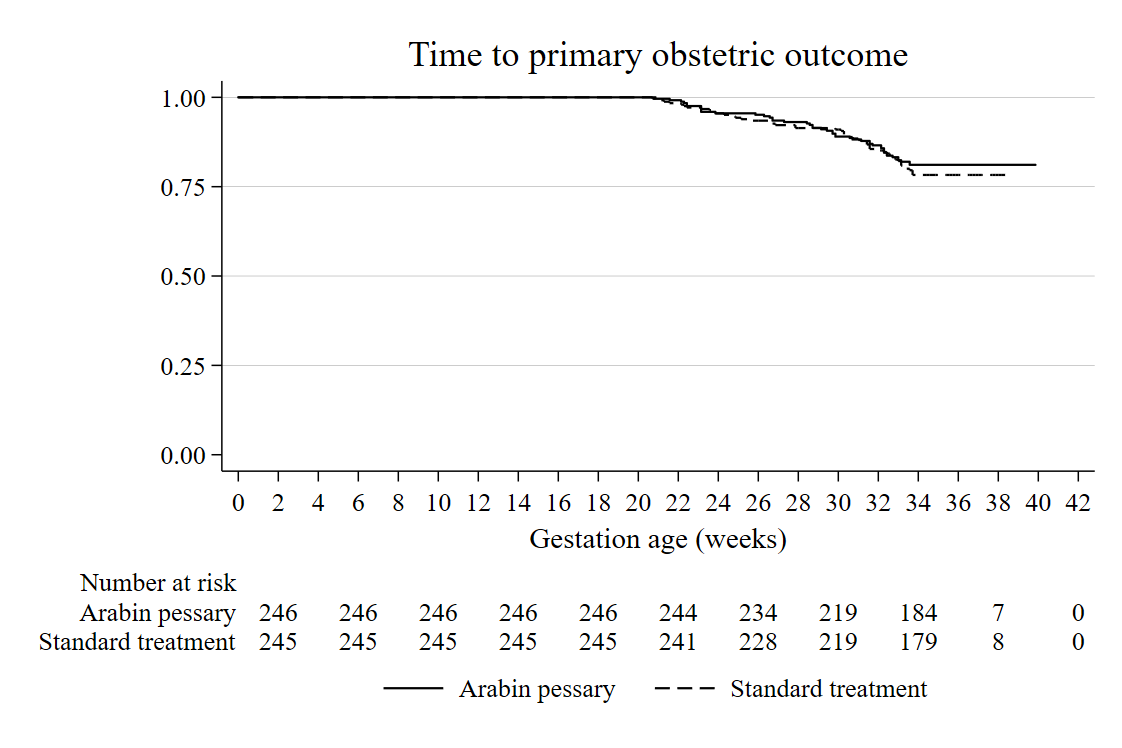

Supplement: S3 Fig — (TIF) [file pmed.1003506.s004.tif]

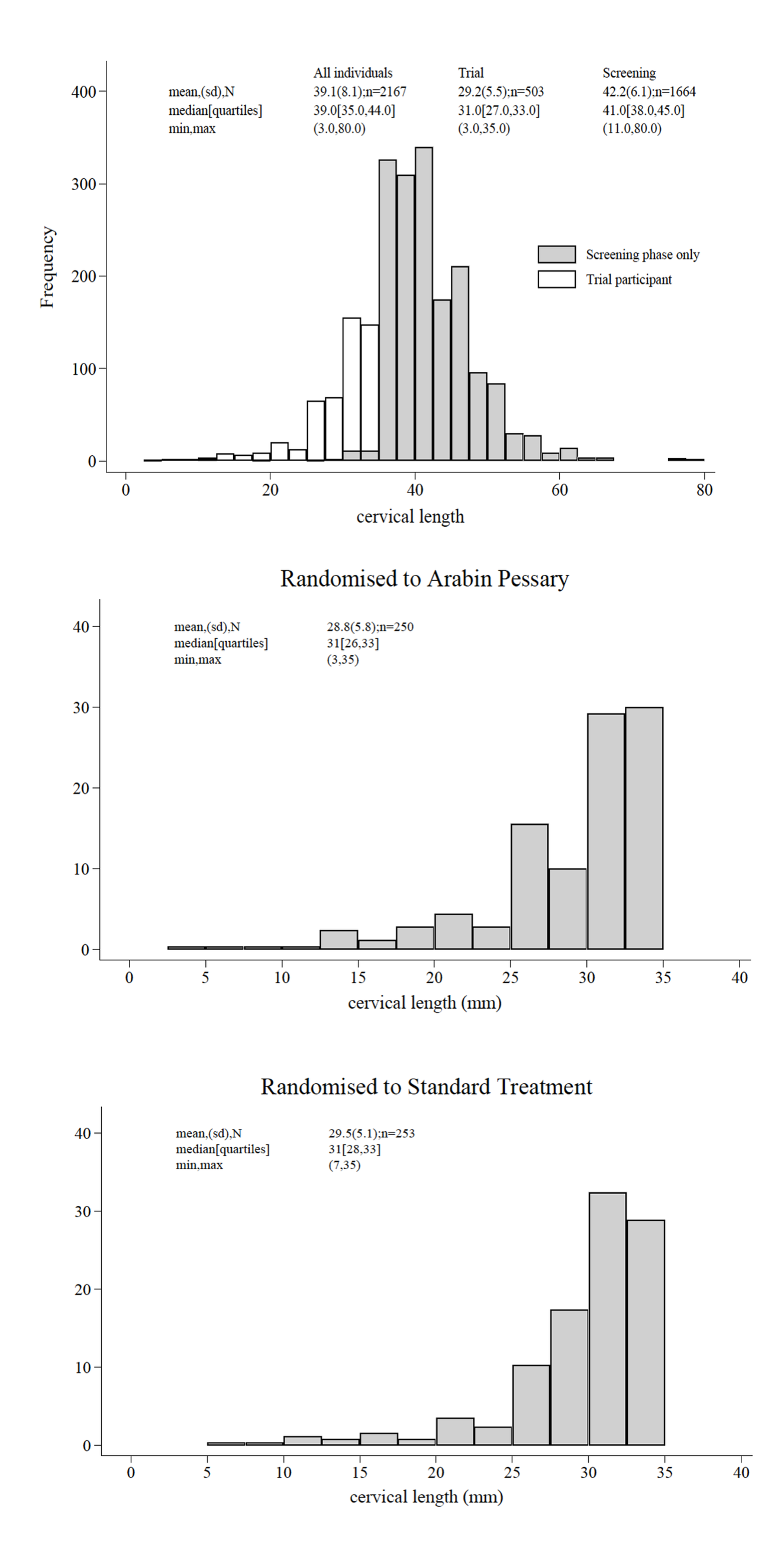

Supplement: S4 Fig — (TIF) [file pmed.1003506.s005.tif]
