# Supplementary material for: Evaluation of the Arabin cervical pessary for prevention of preterm birth in women with a twin pregnancy and short cervix (STOPPIT-2): An open-label randomised trial and updated meta-analysis
Source: PLoS Med. 2021 Mar 29;18(3):e1003506. doi: 10.1371/journal.pmed.1003506 (PMC8041194; doi:10.1371/journal.pmed.1003506)
Supplement: S1 Table — (DOCX) [file pmed.1003506.s006.docx]

**S1 Table Frequency of use of pessary size for first pessary placement in the STOPPIT-2 study.**

| Pessary size (mm) | count |
| --- | --- |
| 70x25x32 | 181 |
| 65x25x32 | 37 |
| 65x25x35 | 9 |
| 70x21x32 | 5 |
| 65x17x32 | 3 |
| 65x21x32 | 2 |
| 70x21x35 | 1 |
| 70x25x35 | 1 |
| 70x30x32 | 1 |
