# Supplementary material for: Evaluation of the Arabin cervical pessary for prevention of preterm birth in women with a twin pregnancy and short cervix (STOPPIT-2): An open-label randomised trial and updated meta-analysis
Source: PLoS Med. 2021 Mar 29;18(3):e1003506. doi: 10.1371/journal.pmed.1003506 (PMC8041194; doi:10.1371/journal.pmed.1003506)
Supplement: S2 Table — (DOCX) [file pmed.1003506.s007.docx]

**S2 Table Safety issues**

p-value from Fisher’s Exact test on subjects· * indicates events reported at the mother level, other events are reported at the baby level

| Type | Arabin pessary  N =250 | Standard treatment  N =253 | p-value from exact test  (Fishers) | p-value from exact test  (Barnard) |
| --- | --- | --- | --- | --- |
| Mother died* | 0/250 (0·0) | 0 / 253 (0·0) | 1·00 |  |
| Any baby death | 22/500 (4·4) | 28/506 (5·5) | 0·39 | 0.53 |
| Intrauterine death – stillbirth  miscarriage | 6/500 (1·2) 12/500 (2·4) | 4/506 (0·8) 16/506 (3·2) | 0·75  0·45 | 0.57  0.53 |
| Neonatal death | 4/500 (0·8) | 8/506 (1·6) | 0·26 | 0.25 |
| Involved or prolonged inpatient maternal hospitalisation* | 11/250 (4·4) | 8/253 (3·2) | 0·64 | 0.53 |
| Involved persistent/significant maternal disability/incapacity* | 0/250 (0·0) | 0/253 (0·0) |  |  |
| Life threatening* | 1/250 (0·4) | 1/253 (0·4) | 1·00 | 1.00 |
| Chorioamnionitis or intrauterine infection* | 12/250 (4·8) | 13/253 (5·1) | 0·84 | 0.89 |
| Congenital anomaly/birth defect* | 1/250 (0·4) | 0/253 (0·0) | 1·00 | 0.53 |
