# Supplementary material for: Evaluation of the Arabin cervical pessary for prevention of preterm birth in women with a twin pregnancy and short cervix (STOPPIT-2): An open-label randomised trial and updated meta-analysis
Source: PLoS Med. 2021 Mar 29;18(3):e1003506. doi: 10.1371/journal.pmed.1003506 (PMC8041194; doi:10.1371/journal.pmed.1003506)
Supplement: S3 Table — (DOCX) [file pmed.1003506.s008.docx]

**S3 Table Experiences of the pessary**

| **Experience of pessary at fitting** | | **n (%)** |
| --- | --- | --- |
| Maternal experience of having device fitted – | |  |
|  | Painless | 36 / 234 (15·4) |
|  | Slightly uncomfortable | 122 / 234 (52·1) |
|  | Uncomfortable | 45 / 234 (19·2) |
|  | Very uncomfortable | 23 / 234 (9·8) |
|  | Worst pain imaginable | 2 / 234 (0·9) |
|  | No response | 6 / 234 (2·6%) |
|  |  |  |
| Clinical team experience of fitting device | |  |
|  | Easy | 158 / 234 (67·5) |
|  | Moderately easy | 44 / 234 (18·8) |
|  | Neither easy nor difficult | 8 / 234 (3·4) |
|  | Difficult | 15 / 234 (6·4) |
|  | Very difficult | 3 / 234 (1·3) |
|  | Impossible | 1 / 234 (0·4) |
|  | No response | 5 / 234 (2·1%) |
|  | |  |
| **Maternal experience of device during pregnancy as reported at 36 week questionnaire** | | n(%) |
|  | |  |
| I could feel the pessary – | |  |
| Never | | 82/139(59·0%) |
| A few times | | 42/139(30·2%) |
| At least once every week | | 3/139(2·2%) |
| Every day | | 7/139(5·0%) |
| All the time | | 5/139(3·6%) |
| No response | | 91/230(39·6%) |
|  | |  |
| I found the pessary uncomfortable | |  |
| Never | | 100/140(71·4%) |
| A few times | | 30/140(21·4%) |
| At least once every week | | 4/140(2·9%) |
| Every day | | 2/140(1·4%) |
| All the time | | 4/140(2·9%) |
| No response | | 90/230(39·1%) |
|  | |  |
| I found the pessary painful | |  |
| Never | | 144/159(90·6%) |
| A few times | | 11/159(6·9%) |
| At least once every week | | 1/159(0·6%) |
| Every day | | 2/159(1·3%) |
| All the time | | 1/159(0·6%) |
| No response | | 71/230(30·9%) |
|  | |  |
| I had vaginal discharge | |  |
| Never | | 7/139(5·0%) |
| A few times | | 20/139(14·4%) |
| At least once every week | | 20/139(14·4%) |
| Every day | | 54/139(38·8%) |
| All the time | | 38/139(27·3%) |
| No response | | 91/230(39·6%) |
|  | |  |
| I had vaginal bleeding | |  |
| Never | | 116/139(83·5%) |
| A few times | | 20/139(14·4%) |
| At least once every week | | 1/139(0·7%) |
| All the time | | 2/139(1·4%) |
| No response | | 91/230(39·6%) |
|  | |  |
| I had to use panty liners/sanitary towels because of the vaginal discharge/bleeding | |  |
| Never | | 26/140(18·6%) |
| A few times | | 21/140(15·0%) |
| At least once every week | | 12/140(8·6%) |
| Every day | | 37/140(26·4%) |
| All the time | | 44/140(31·4%) |
| No response | | 90/230(39·1%) |
|  | |  |
| The vaginal discharge was | |  |
| The same as before the pessary | | 16/132(12·1%) |
| A little more than before the pessary | | 41/132(31·1%) |
| A lot more than before the pessary | | 75/132(56·8%) |
| No response | | 98/230(42·6%) |
|  | |  |
| The vaginal discharge was | |  |
| The same as in my last pregnancy | | 5/116(4·3%) |
| A little more than in my last pregnancy | | 16/116(13·8%) |
| A lot more than in my last pregnancy | | 33/116(28·4%) |
| This is my first pregnancy | | 62/116(53·4%) |
| No response | | 114/230(49·6%) |
|  | |  |
| The vaginal bleeding was | |  |
| The same as before the pessary | | 30/44(68·2%) |
| A little more than before the pessary | | 7/44(15·9%) |
| A lot more than before the pessary | | 7/44(15·9%) |
| No response | | 186/230(80·9%) |
|  | |  |
| The vaginal bleeding was | |  |
| The same as in my last pregnancy | | 12/50(24·0%) |
| A little more than in my last pregnancy | | 2/50(4·0%) |
| A lot more than in my last pregnancy | | 3/50(6·0%) |
| This is my first pregnancy | | 33/50(66·0%) |
| No response | | 180/230(78·3%) |
|  | |  |
| **Experience of device removal** | | n (%) |
| Maternal experience of having device removed– | |  |
| Painless | | 45/184(24·5%) |
| Slightly uncomfortable | | 49/184(26·6%) |
| Uncomfortable | | 36/184(19·6%) |
| Very uncomfortable | | 43/184(23·4%) |
| Worst pain imaginable | | 11/184(6·0%) |
| No response | | 46 / 230 (20·0%) |
|  | |  |
| Clinical team experience of device removal | |  |
| Easy | | 103/172(59·9%) |
| Moderately easy | | 30/172(17·4%) |
| Neither easy nor difficult | | 15/172(8·7%) |
| Difficult | | 21/172(12·2%) |
| Very difficult | | 3/172(1·7%) |
| No response | | 58 / 230 (25·2%) |
