# Supplementary material for: Evaluation of the Arabin cervical pessary for prevention of preterm birth in women with a twin pregnancy and short cervix (STOPPIT-2): An open-label randomised trial and updated meta-analysis
Source: PLoS Med. 2021 Mar 29;18(3):e1003506. doi: 10.1371/journal.pmed.1003506 (PMC8041194; doi:10.1371/journal.pmed.1003506)
Supplement: S4 Table — (DOCX) [file pmed.1003506.s009.docx]

**S4 Table**

| **Outcomes for specified cervical length** | **Spontaneous birth before 34 weeks gestation** | |
| --- | --- | --- |
| Cervical Length 35mm | Yes | No |
| Less than equal to threshold | True Positives (TP) 58 | False Positives (FP) 223 |
| Greater than threshold | False Negatives (FN) 146 | True Negatives (TN) 1453 |
| Total | 204 | 1676 |
| Sensitivity | 0·28(0·22,0·35) | |
| Specificity | 0·87(0·85, 0·88) | |
| Positive likelihood ratio | 2·14(1·67, 2·74) | |
| Negative likelihood ratio | 0·83(0·76, 0·90) | |
|  |  | |
| **Outcomes for specified cervical length** | **Spontaneous birth before 34 weeks gestation** | |
| Cervical Length 30mm | Yes | No |
| Less than equal to threshold | True Positives (TP) 35 | False Positives (FP) 88 |
| Greater than threshold | False Negatives (FN) 169 | True Negatives (TN) 1588 |
| Total | 204 | 1676 |
| Sensitivity | 0·17(0·12, 0·22) | |
| Specificity | 0·95(0·94, 0·96) | |
| Positive likelihood ratio | 3·27(2·27, 4·70) | |
| Negative likelihood ratio | 0·87(0·82, 0·93) | |
|  |  |  |
| **Outcomes for specified cervical length** | **Spontaneous birth before 34 weeks gestation** | |
| Cervical Length 28mm | Yes | No |
| Less than equal to threshold | True Positives (TP) 25 | False Positives (FP) 47 |
| Greater than threshold | False Negatives (FN) 179 | True Negatives (TN) 1630 |
| Total | 204 | 1676 |
| Sensitivity | 0·12(0·08, 0·17) | |
| Specificity | 0·97(0·96, 0·98) | |
| Positive likelihood ratio | 4·37(2·75, 6·94) | |
| Negative likelihood ratio | 0·90(0·86, 0·95) | |
|  |  | |
|  |  | |
| **Outcomes for specified cervical length** | **Spontaneous birth before 34 weeks gestation** | |
| Cervical length 25mm |  |  |
| Less than equal to threshold | True Positives (TP) 20 | False Positives (FP) 21 |
| Greater than threshold | False Negatives (FN) 184 | True Negatives (TN) 1655 |
| Total | 204 | 1676 |
| Sensitivity | 0·10(0·06, 0·14) | |
| Specificity | 0·99(0·98, 0·99) | |
| Positive likelihood ratio | 7·82(4·32, 14·18) | |
| Negative likelihood ratio | 0·91(0·87, 0·96) | |
|  |  |  |
| **Outcomes for specified cervical length** | **Spontaneous birth before 34 weeks gestation** | |
| Cervical length 20mm |  |  |
| Less than equal to threshold | True Positives (TP) 10 | False Positives (FP) 9 |
| Greater than threshold | False Negatives (FN) 194 | True Negatives (TN) 1667 |
| Total | 204 | 1676 |
| Sensitivity | 0·05 (0·02, 0·08) | |
| Specificity | 1.00 (0·99, 1.00) | |
| Positive likelihood ratio | 9·13 (3·75, 22·20) | |
| Negative likelihood ratio | 0·96 (0·927, 0·987) | |
